# Spatial analysis and temporal trends of porcine reproductive and respiratory syndrome in Denmark from 2007 to 2010 based on laboratory submission data

**DOI:** 10.1186/s12917-015-0617-0

**Published:** 2015-12-21

**Authors:** Ana Carolina Lopes Antunes, Tariq Halasa, Klara Tølbøl Lauritsen, Charlotte Sonne Kristensen, Lars Erik Larsen, Nils Toft

**Affiliations:** Section for Epidemiology, National Veterinary Institute, Technical University of Denmark, Bülowsvej 27, 1870 Frederiksberg C, Denmark; Section for Diagnostic and Scientific Advice, National Veterinary Institute, Technical University of Denmark, Bülowsvej 27, 1870 Frederiksberg C, Denmark; Danish Pig Research Centre, SEGES P/S, Vinkelvej 11, 8620 Kjellerup, Denmark; Section for Virology, National Veterinary Institute, Technical University of Denmark, Bülowsvej 27, 1870 Frederiksberg C, Denmark

**Keywords:** PRRSV, Laboratory submission, Spatiotemporal, Seroprevalence, Serocovertion rate

## Abstract

**Background:**

Porcine reproductive and respiratory syndrome (PRRS) has been a cause for great concern to the Danish pig industry since it was first diagnosed in 1992. The causative agent of PRRS is an RNA virus which is divided into different genotypes. The clinical signs, as well as its morbidity and mortality, is highly variable between herds and regions. Two different genotypes of PRRS virus (PRRSV) are found in Denmark: type 1 and type 2. Approximately 40 % of Danish swine herds are seropositive for one or both PRRSV types. The objective of this study was to describe the temporal trend and spatial distribution of PRRSV in Danish swine herds from 2007 to 2010, based on type-specific serological tests from the PRRS surveillance and control program in Denmark using the results stored in the information management system at the National Veterinary Institute, Technical University of Denmark (DTU Vet).

**Results:**

The average monthly seroprevalence of PRRSV type 1 was 9 % (minimum of 5 %; maximum of 13 %) in breeding herds, and 20 % (minimum of 14 %; maximum of 26 %) in production herds; PRRSV type 2 had an average seroprevalence of 3 % (minimum of 1 %; maximum of 9 %) in breeding herds and of 9 % (minimum of 5 %; maximum of 13 %) within production herds. The seroconversion rate followed a similar and consistent pattern, being higher for type 1 than for type 2 for both PRRSV types. Regarding the spatiotemporal results, the relative risk distribution maps changed over time as a consequence of the changes in PRRSV seroprevalence, suggesting a general decline in the extent of areas with higher relative risk for both type 1 and 2. Local spatial analysis results demonstrated the existence of statistically significant clusters in areas where the relative risk was higher for both herds.

**Conclusions:**

PRRSV type 1 seroprevalence was constantly higher than for PRRSV type 2 in both herd types. Significant spatial clusters were consistently found in Denmark, suggesting that PRRSV is endemic in these areas. Furthermore, relative risk distribution maps revealed different patterns over time as a consequence of the changes in seroprevalence.

**Electronic supplementary material:**

The online version of this article (doi:10.1186/s12917-015-0617-0) contains supplementary material, which is available to authorized users.

## Background

Porcine reproductive and respiratory syndrome (PRRS) causes significant financial losses for the pig industry in Europe, United States (US) and Asia [1-5].

The causative agent of PRRS is an RNA virus [6, 7], the PRRS virus (PRRSV), which is divided into genotypes: type 1 and type 2, previously known as European and North American strains, respectively [8]. The severity of the diseases is highly variable between herd as a result from immunological factors, herd management and the pathogenicity resulting in different clinical signs, morbility and mortality rates [9, 10].

The first Danish case of PRRSV type 1 was diagnosed in March 1992 in a sow herd located in southern Denmark [11]. A voluntary PRRSV control program was established in 1996 by the Federation of Danish Pig Producers and Slaughterhouses in order to reduce the spread of the virus. Initially, a national serological screening based on an DTU Vet (National Veterinary Institute, Technical University of Denmark) “in-house” Blocking Enzymed-Linked Immunosorbent Assay (ELISA) and Immunoperoxidase monolayer assay (IPMA) was carried out, which demonstrated that the seroprevalence of PRRSV type 1 in Danish herds was 33 % [12]. This screening did not reveal the presence of PRRSV type 2. The second step included vaccination of 1100 herds with a modified-live PRRSV type 2 vaccine, between 1 July and 1 October 1996. The vaccine was approved by the Danish Health authorities from 1 July 1996 and licensed for use in pigs between 3 and 18 weeks old. However, this vaccine had already been used in October 1995 to vaccinate all boars entering artificial insemination stations [13]. This procedure was performed in quarantine units with special permission from the Danish authorities. Following approval in 1996, the vaccination was not only carried out in PRRSV seropositive herds, but also in many herds that had no clinical symptoms of PRRS.

In 1997, PRRSV type 2 was isolated for the first time in Denmark from fetuses, dead piglets and sows, suggesting transplacental infection had occurred after PRRSV infection of pregnant sows. In addition, non-vaccinated Danish herds previously uninfected with PRRSV type 1 had become infected with the vaccine-like PRRSV. The PRRSV type 2 virus was also spread from artificial insemination centers in semen, by introducing vaccinated animals to herds, and by airborne transmission to PRRS-free and non-vaccinated herds [13].

Despite disease control efforts in Denmark, PRRS continues to contribute towards the economic losses associated with mortality in piglets, respiratory problems in growers and finishers, and reproductive problems in sows. Furthermore, previously full sequencing of PRRSV type 1 and type 2 [14, 15], demonstrated a high variance in several genomic regions in the PRRSV type 1 strains circulating in Denmark, further complicating the control of the disease. Currently, the between-herd seroprevalence of PRRSV in the Danish pig population is considered to be around 40 %, based on the number of herds with a known status (unpublished data). Spatial and temporal analysis can be used to identify the location, shapes and sizes of potential diseases outbreaks [16].

The spatiotemporal description of PRRS based on laboratory data might help decision makers to re-evaluate their conclusions on the spread of the disease and assess the efficiency of the implemented control strategies. DTU Vet was the only laboratory in Denmark to perform serological tests for PRRS virus from 2007 to 2010. Using only the data from 2007 to 2010 would therefore allow us to study the spatiotemporal occurrence of PRRS. This analysis will allow us to characterize changes in the PRRSV seroprevalence and seroconversion rate, and to assess the spatial distribution of PRRSV seropositive herds, facilitating control of the disease on local and regional basis, e.g. by changing management routines, trade customs etc. and make a descriptive analysis and find patterns, clusters, etc to make help prioritize funds for controlling these diseases.

The objective of the present study was to describe the temporal trend and spatial distribution of both PRRSV types in Danish breeding and production herds from 2007 to 2010.

## Methods

### Data description

The Specific Pathogen Free (SPF) System was implemented in Denmark in 1971. It is a voluntary health program with established rules for monitoring Enzootic pneumonia, Porcine pleuropneumonia, Swine dysentery, Atrophic rhinitis, PRRS, mange and lice [17]. This program is primarily based on serological testing performed on a regular basis according to the herd type: the breeding herds (including nucleus and multiplier herds) are tested on a monthly basis and are classified as “red” herds; the production herds (including farrow-to-finisher and finisher herds) are tested every 12 months and classified as “blue” herds. The “red” and “blue” are designation used within the SPF system to classify the herds according to its herd health status. For each testing is necessary to take individual blood samples from 10 animals (5 gilts and 5 sows) and from 20 animals randomly selected within the herd for the red and blue herds respectively (personal communication, C.S. Kristensen, 2014). The SPF herds represent about 40 % of all Danish swine herds, but since many large farms are enrolled, 73 % of the Danish sows are included [18].

The laboratory submissions are requested according to the SPF status of the herd. For non-SPF herds, the veterinarians can decide which serological test to request, and at what interval. The outcome of this decision will depend on the overall objective and the costs associated with the different serology tests.

Laboratory submissions stored in the DTU Vet information management system in the period from 1 January 2007 to 31 December 2010 were extracted. Each laboratory submission consisted of individual blood samples collected from the same herd on the same day. Only submissions with between 2 and 60 individual blood samples tested by serological tests ELISA and/or IPMA for one or both PRRSV strains were included in the analysis. A total of 27,854 laboratory submissions tested for PRRS at the National Veterinary Institute were included in the analysis, representing a total of 879,327 serological tests performed on 404,029 individual blood samples collected from a total of 4702 Danish swine herds.

The laboratory submissions were merged with the SPF system database in order to classify the herds into breeding and production herds. All red herds in the SPF system database were classified as breeding herds (*N* = 264); the remaining herds (blue SPF herds and non-SPF herds) were classified as production herds (*N* = 4438). The herd classification was year-specific since the SPF health status can change over time.

### Ethics approval

The study was conducted using surveillance data and did not involve experiments on animals. The serum samples used for the study were obtained from blood samples voluntarily collected for monitoring PRRS. From an ethical perspective, all of the material collected and used as part of this study was outside the scope of Directive 2010/63.

### PRRSV status

The herd PRRSV status in each laboratory submission was defined based on the cut-off for individual blood tests, in addition to the herd-level cut-off, which establishes the proportion of PRRSV seropositive samples (i.e. animals) within the herd. This approach was performed due to the recognized cross-reactivity between serological tests for the two PRRSV types [19], and the possible co-existence of PRRSV type 1 and 2 within herds [20].

For herds with more than one submission per month, the latest submission within the month was used to classify the herd.

### Individual blood samples classification

At DTU Vet, in-house ELISAs and IPMAs were used to test for PRRSV antibodies, enabling us to distinguish between PRRSV type 1 and PRRSV type 2 specific antibodies.

The blocking ELISAs were performed according to [19, 21]; ELISA plates were separately coated with either PRRSV type 1 or 2 antigens, and the individual test serum samples were added to both the type 1 and the type 2 ELISA-plates. After incubation with the samples, biotinylated polyclonal swine-IgG directed against either PRRSV type 1 or 2, respectively, was added to the plates. For final development, peroxidase conjugated streptavidine and TMB were used, and colorimetric reactions were then measured based on optical density (OD). Results were considered positive if the OD% ≤44. Both ELISAs were run in parallel for the same sample and if the test result was positive for at least one type, the type 1/type 2 ratio was determined based on the obtained OD values in order to distinguish between the two PRRSV types. Ratios below 1.3 indicated the presence of type 1 PRRSV whereas ratios above 1.9 was an indication of type 2 [19]. Ratios between these values did not allow for distinction between the two PRRSV types.

The IPMA technique is described by [11]. In summary, the IPMA plates were prepared with MARC-145 cell lines, fixed with either PRRSV type 1 or 2 [21]. These plates were then incubated with serial sample dilutions from 50 to 6250. The enzyme peroxidase was used to catalyse a chemical reaction to color PRRSV specifically stained cells, and the plates were examined under a microscope. Specific staining of infected cells indicated the presence of PRRSV antibodies.

Serological tests with missing results in the database were excluded from the analysis (*N* = 6202).

Each individual blood sample was classified as PRRSV type 1 seropositive, PRRSV type 2 seropositive, PRRSV type 1 and 2 seropositive or seronegative according to the following criteria:

- Samples only tested by IPMA were classified according to [21];
- Samples tested by both ELISAs were classified based on the ratio type 1/type 2 according to [19];
- Samples with ratios between 1.3 and 1.9, were classified as both PRRSV type 1 and 2 seropositive;
- For samples tested by ELISA and IPMA, the IPMA results were prioritized in order to identify the PRRSV type;
- Samples tested only against one PRRSV strain by ELISA or IPMA were classified based only on those results.

### Herd-level PRRSV classification

The herd-level PRRSV status was defined based on the number of PRRSV seropositive samples as suggested by [22]. The number of individual blood samples tested by ELISA and IPMA to classify the herd PRRS status varied according to the total number of individual blood samples tested per herd.

For herds with animals tested for both strains by IPMA, the classification was made following a comparison of titers in IPMA-PRRSV type 1 and IPMA-PRRSV type 2 in each individual sample. Herds were defined as PRRSV type 2 seropositive if the number of individual blood samples with IPMA-PRRSV type 2 ≥ IPMA-PRRSV type 1 per submissions [number of individual blood samples tested per submission] was equal or higher than 2 [2–5], 3 [6–15], 4 [16–18], 5 [29–35], 6 [36–45] and 7 [46–60]. Herds were defined PRRSV type 1 seropositive if the number of individual blood samples with IPMA-PRRSV type 1 > IPMA-PRRSV type 2 was equal or higher than 2 [2–7], 3 [8–15], 4 [16–29], 5 [30–45] and 6 [46–60]. For those submissions where IPMA was used to test for only one PRRSV type, the herds were considered to be PRRSV type 2 seropositive when IPMA-PRRSV type 2 titers ≥ 1250 was equal or higher than 2 [2–5], 3 [6–10], 4 [11–15], 5 [16–21], 6 [22–28], 7 [29–36], 9 [37–46] and 11 [47–60]. In addition, the cut-off points to classify herds as PRRSV type 1 seropositive were 2 [2–5], 3 [6–10], 4 [11–15], 5 [16–22], 6 [23–29], 7 [30–36], 8 [37–44], 9 [45–53] and 10 [54–61] IPMA-PRRSV type 1 titers ≥ 1250 in individual samples.

For laboratory submissions with individual blood samples tested only by ELISA, the herds were classified as PRRSV type 1 seropositive if they had at least 2 [2–19], 3 [20–39], 4 [40–59] or 5 [60] individual blood samples with a ratio <1.2. If the herds had at least 2 samples with a ratio ≥ 2 at any sample size, these herds were classified as PRRSV type 2 seropositive.

For a herd to be classified as PRRSV seronegative, all individual blood samples must test seronegative for both PRRSV types in both tests (ELISA and IPMA).

### Statistical analysis

#### PRRSV seroprevalence in herds submitting samples

The seroprevalence of PRRSV type 1 and 2 in herds submitting samples was calculated on a monthly basis, where the number of PRRSV positive herds was divided by the total number of herds tested for PRRSV in that specific month.

#### Seroconversion rate in breeding herds

According to 23], PRRSV antibody titers reach the lower limits of detection at around 324 days post-inoculation (PI). Therefore, the breeding herds were classified as newly PRRSV seropositive if they had been seronegative in the previous 12 months. The number of new positive herds was modelled assuming a negative binomial distribution according to the following model:

1 $$ Y\sim \mu + offset\ \left( \log (tar)\right) $$

where *Y* is the number of new positive herds per month from January 2008 to December 2010, *μ* is the intercept of the model and *tar* is the average time at risk in the previous 12 months. The average time at risk was calculated for each month based on the average number of previous months in which the herds were PRRS seronegative (i.e. classified as susceptible).

#### Herd identification

The herd identification number was used to obtain the geographic coordinates (UTM EUREF89, zone 32) from the CHR (Central Husbandry Register) database. Herds with missing location data (*N* = 107) were omitted from the spatial analysis.

#### PRRSV relative risk maps

PRRSV relative risk maps were made to facilitate visualization of the spatial distribution of PRRSV type 1 and 2 seropositive and seronegative herds biannually from 2007 to 2010.

The odds of a herd at a given location *c* being PRRS positive were calculated as p(*c*) = λ_1_ (*c*) / (λ_1_ (*c*) + λ_0_ (*c*)), where λ_1_ and λ_0_ are the intensity functions of positive and negative herds respectively. The risk surfaces were created by calculating the ratio of intensity functions for positive and negative herds on a grid of 2 × 2 km cells. The kernel smoothing surfaces were calculated based on a Gaussian model [24]; no edge-correction was performed.

The specification of the bandwidth is more important than the choice of kernel function [25]. Therefore, the median of specific biannual bandwidths were calculated for each PRRSV type and used to perform kernel smoothing, in order to identify any temporal differences.

#### Cluster analysis

Retrospective Space Scan Statistics [26] were used to identify local spatial clusters of PRRSV type 1 and type 2 seropositive herds biannually from 2007 to 2010. This method has been used in veterinary medicine to identify PRRSV outbreaks in United States [27] and Canada [28]. The Bernoulli model was used since the herds were classified as either PRRSV type 1 and 2 seropositive (cases) or seronegative (controls). The scanning window was circular and no overlapping clusters were permitted. The analysis was repeated five times using different maximum population sizes (i.e. herds) at risk, including 5, 15, 25, 35 and 50 %. The *p*-value was obtained using 999 Monte Carlo simulations and a 5 % significance level was used based on a likelihood ratio test.

All analyses were performed in R version 3.1.1 [29]. Kernel smoothing densities were made using the’sm package’ [30] for estimating the bandwidth and’spatialkernel package’ [31] for kernel estimation. Spatial cluster analysis was based on SatScan version 9.3.1 32].

## Results

### Data description

The total number of herds, laboratory submissions and blood samples tested per year during the period from January 2007 to December 2010 are listed in Table 1. On average, 2776 production and 230 breeding herds were tested annually; the median number of annual submissions was 12 for breeding herds and 1 for production herds. The average time between two consecutive submissions was 1 month (maximum of 37) for breeding herds and 11.33 months (minimum of 1 and maximum of 46) for production herds. The descriptive statistics of PRRS serological diagnostic tests performed are described in Table 2.

**Table 1:** Descriptive statistics by frequency of laboratory submissions sent to DTU Vet laboratory for testing PRRSV during the period from 2007 to 2010 for breeding (Breed) and production (Prod) herds. Each laboratory submission consisted of individual blood samples collected from the same herd on the same day

Year	2007		2008		2009		2010	
Herd type	Breed	Prod	Breed	Prod	Breed	Prod	Breed	Prod
Total number of tested herds	237	2982	233	2729	228	2720	220	2673
Median number of submissions per herd (Q1 – Q3)	12 (12–13)	1 (1–1)	12 (12–13)	1 (1–1)	12 (12–13)	1 (1–1)	12 (12–13)	1 (1–1)
Total number of samples	31,505	73,561	33,430	69,233	30,572	67,640	33,420	64,668
Median number of samples per herd (Q1–Q3)	10 (10–15)	20 (17–20)	10 (10–15)	20 (16–20)	10 (10–10)	20 (15–20)	10 (10–15)	20 (15–20)

**Table 2:** Descriptive statistics by frequency (N) and percentage (%) of PRRS serological diagnostic tests performed from 2007 to 2010

Year		2007		2008		2009		2010	
	Serological test	*N*	%	*N*	%	*N*	%	*N*	%
Total number of serological tests performed	ELISA-type 1	101,925	44.1	100,172	44.2	95,133	44,7	94,493	45.2
	ELISA-type 2	101,924	44.1	100,174	44.2	95,133	44.7	94,495	45.2
	IPMA-type 1	14,307	6.2	14,426	6.4	12,421	5.8	10,830	5.2
	IPMA-type 2	12,804	5.5	11,775	5.2	10,225	4.8	9040	4.3
Total number of samples		105,066	-	102,663	-	98,212	-	98,088	-
Number of samples only tested by:	ELISA-type 1	1	<0.01	1	<0.01	0	0.00	0	0.00
	ELISA-type 2	0	0.00	3	<0.01	0	0.00	0	0.00
	IPMA-type 1	783	0.8	605	0.6	1021	1.0	1095	1.1
	IPMA-type 2	402	0.4	168	0.2	532	0.5	784	0.8
Number of samples tested by doubled ELISA		89,529	85.2	87,156	84.9	84,569	86.1	85,659	87.3
Number of samples tested by ELISA and IPMA		12,395	11.8	13,015	12.7	10,564	10.8	8837	9.0

**Fig. 1:**
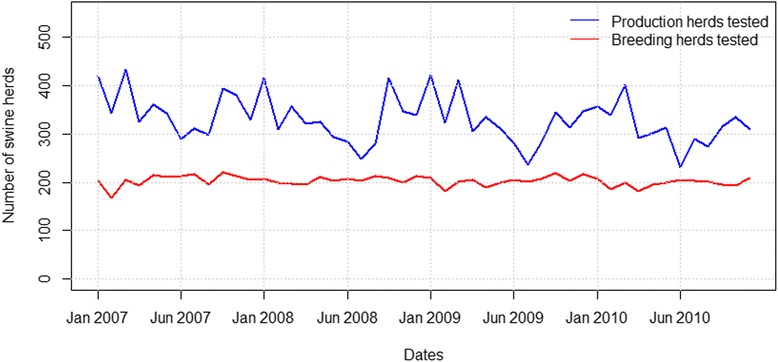
Number of production and breeding herds tested for PRRSV per month from January 2007 to December 2010

The total number of breeding herds submitting samples on a monthly basis between 2007 and 2010 did not vary from year to year. In contrast, the total number of tested production herds followed a seasonal trend (Fig. 1). In general, the number of positive herds followed the same trend as the total number of herds tested. The number of herds testing seropositive was higher for PRRSV type 1 than for PRRSV type 2. This applied to both production and breeding herds, the only exceptions being in April 2007 and June 2010, when the number of PRRSV type 2 seropositive production herds increased to the same value as PRRSV type 1 seropositive production herds.

No herds were classified as positive for both PRRSV types simultaneously in the same month.

**Fig. 2:**
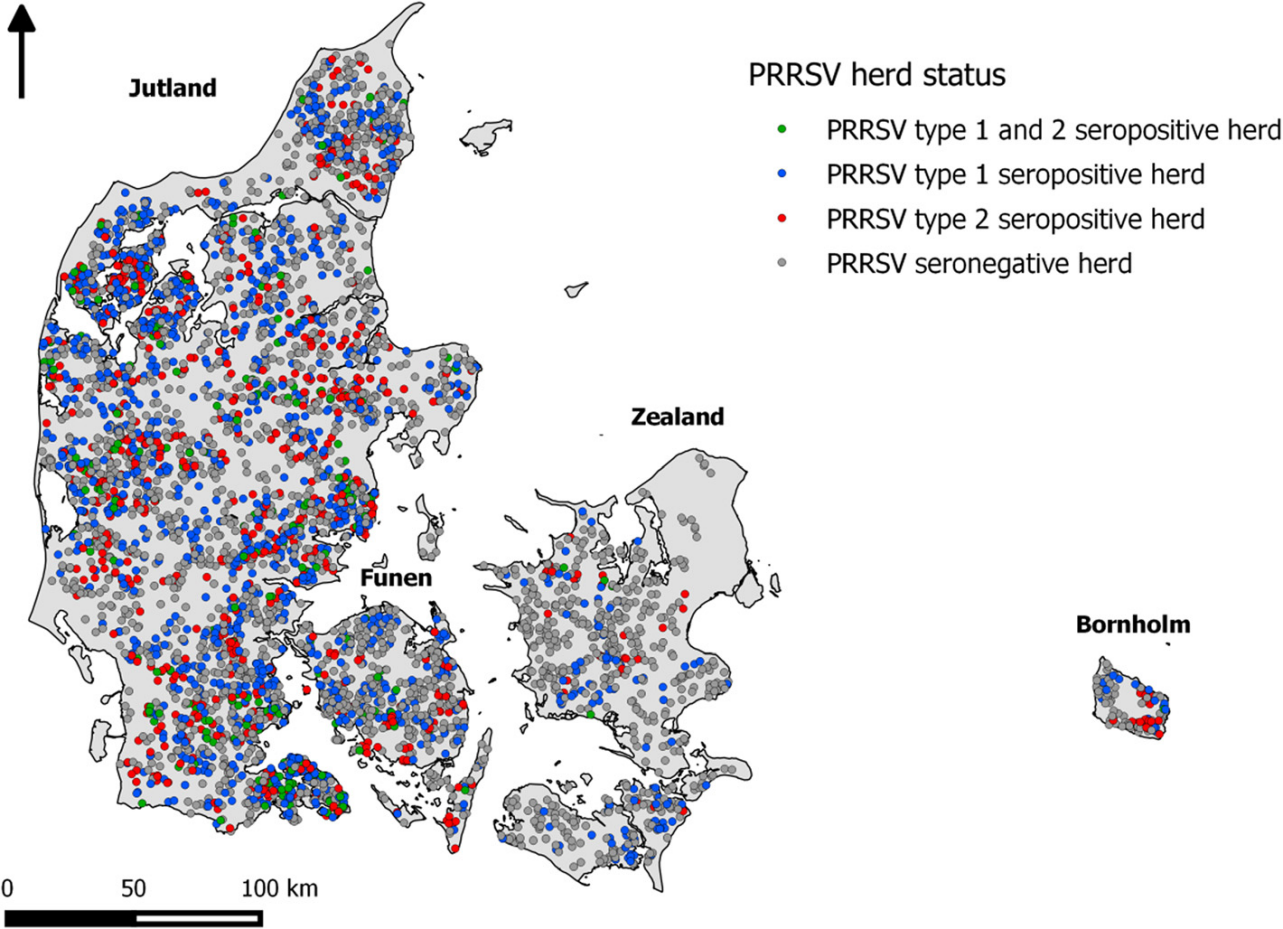
PRRSV herd status distribution from 2007 to 2010, including only herds submitting samples. Herds were classified as PRRS seropositive if they were positive during a minimum of 1 month between 2007 and 2010; herds classified as seropositive for both strains during this period were labeled in green; negative herds (*grey*) were not classified as PRRS positive during the period of study

Figure 2 shows the distribution of all herds tested for PRRSV based on serology from 2007 to 2010. The majority of these herds were located in Jutland, reflecting the higher pig density in this region.

### PRRSV seroprevalence

**Fig. 3:**
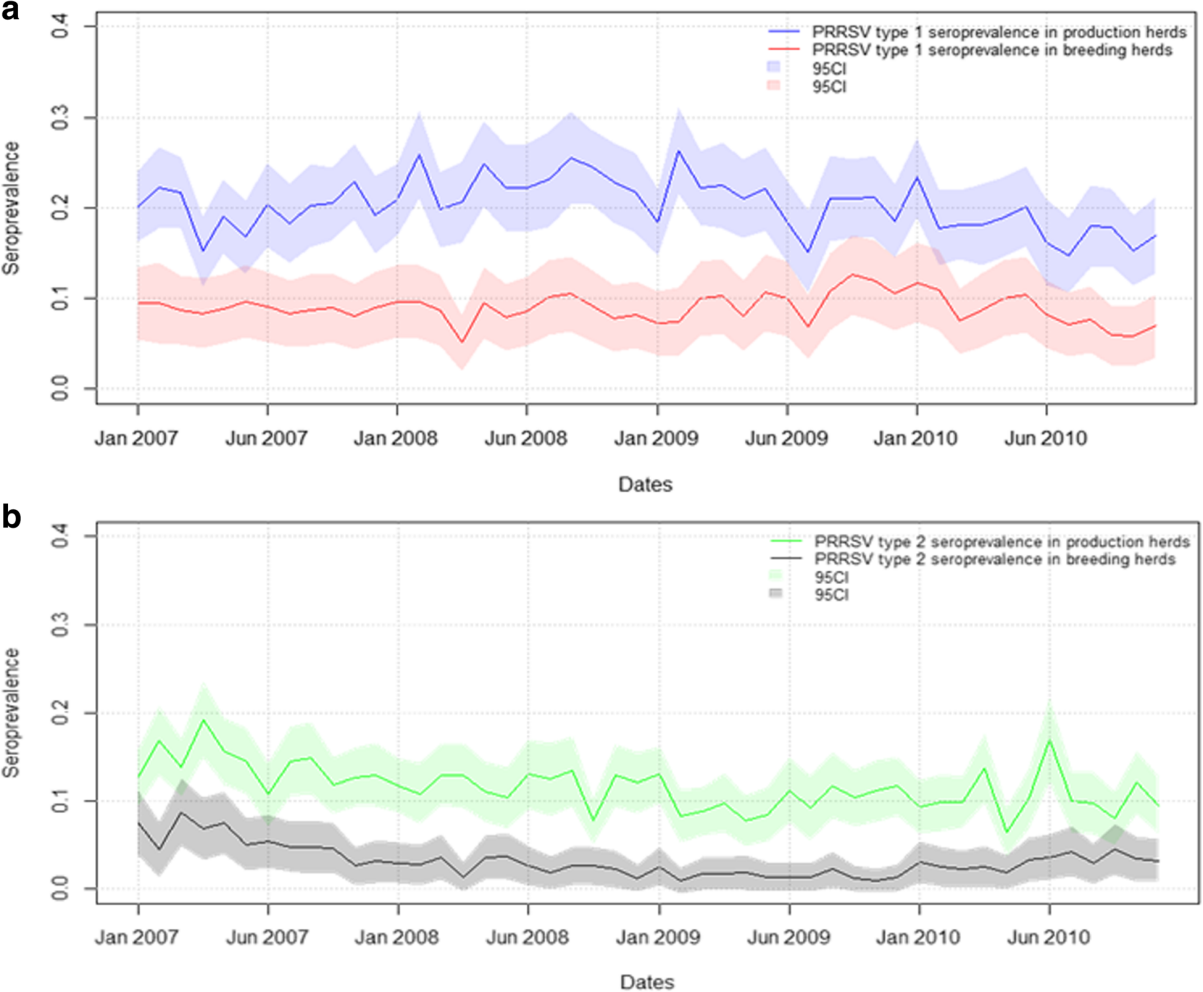
Monthly PRRSV seroprevalence in Danish pig herds. The figure illustrated the monthly PRRSV type 1 (**a**) and type 2 (**b**) seroprevalence in production and breeding herds from January 2007 to December 2010

The apparent PRRSV seroprevalence in tested herds appears to be higher for PRRSV type 1 than for PRRSV type 2 from 2007 to 2010 (Fig. 3). There appeared to be an overall decrease in the seroprevalence for both PRRSV types (though this was not tested for statistical significance). The monthly average PRRSV type 1 seroprevalence was 0.09 (minimum of 0.05; maximum of 0.13) in breeding herds and 0.20 (minimum of 0.14; maximum of 0.26) in production herds; PRRSV type 2 had an average of 0.03 (minimum of 0.01; maximum of 0.09) in breeding herds and 0.09 (minimum of 0.05; maximum of 0.13) in production herds.

### PRRSV seroconversion rate in breeding herds

**Fig. 4:**
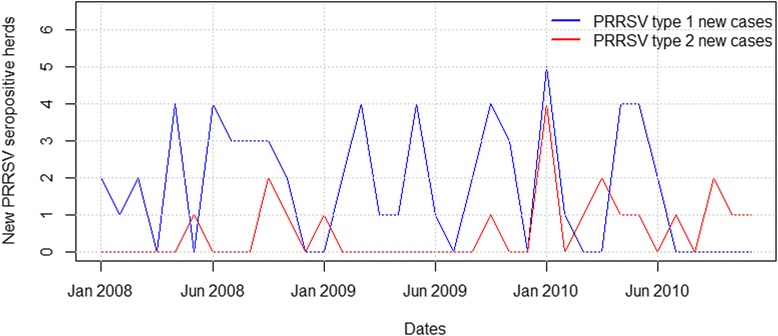
Number of new PRRSV type 1 and 2 seropositive breeding herds from 2008 to 2010

The total number of new PRRSV seropositive breeding herds per month is presented in Fig. 4. The monthly seroconversion rate followed a constant pattern for both PRRSV types, being higher for type 1 (average of 0.65 herds per 100 herds) than type 2 (average of 0.21 herds per 100 herds).

### Smoothed relative risk surfaces

**Fig. 5:**
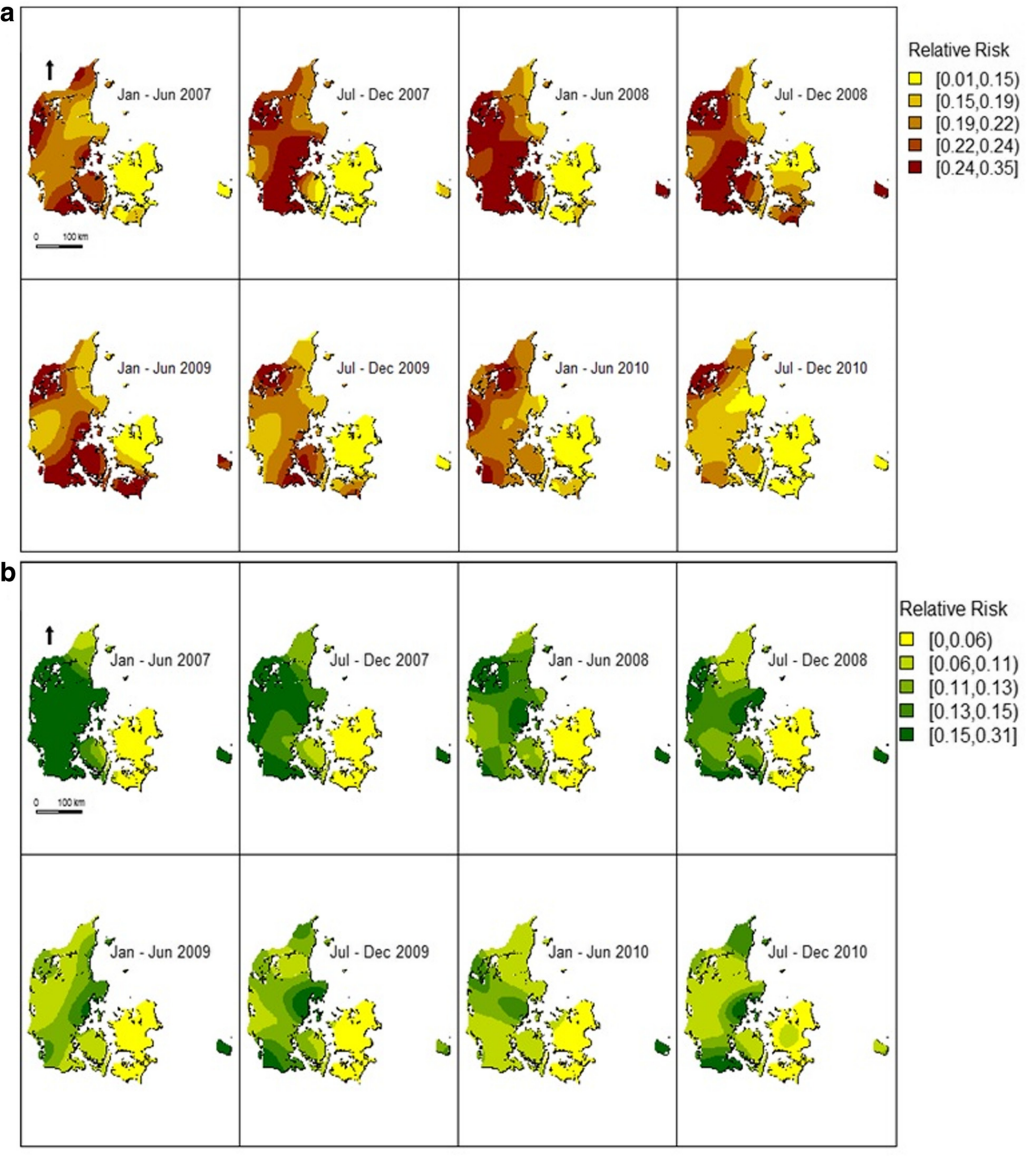
Biannually smoothed relative risk surfaces in Denmark from 2007 to 2010. Smoothed surfaces of the probability of swine herds being PRRSV type 1 (**a**) and type 2 (**b**) seropositive (relative risk) at a given location biannually during the period 2007 to 2010. Legend was defined based on 20 % quantiles

The smoothed relative risk surface of the probability of swine herds being positive for both PRRS-strains changed spatiotemporally (Fig. 5). The median values for the biannual bandwidths were *h* = (29,576.49; 31,069.79) and *h* = (30,885.97; 31,401.67) for PRRSV type 1 and 2, respectively.

In general, the extent of areas with higher relative risk decreased from 2007 to 2010 for both PRRSV types.

Regarding PRRSV type 1 relative risk distribution between July 2007 and December 2008, the areas with the highest relative risk were located in the west of Denmark. During the remaining periods, the same areas covered a smaller geographic area and were located in the northwestern and the southwestern parts of Denmark.

The overall relative risk was lower for PRRSV type 2 when compared to PRRSV type 1. In this case, the highest relative risk areas had a larger extent in 2007, which later decreased. In the following years, these areas remained in the western part of the country.

### Spatial cluster analysis

**Fig. 6:**
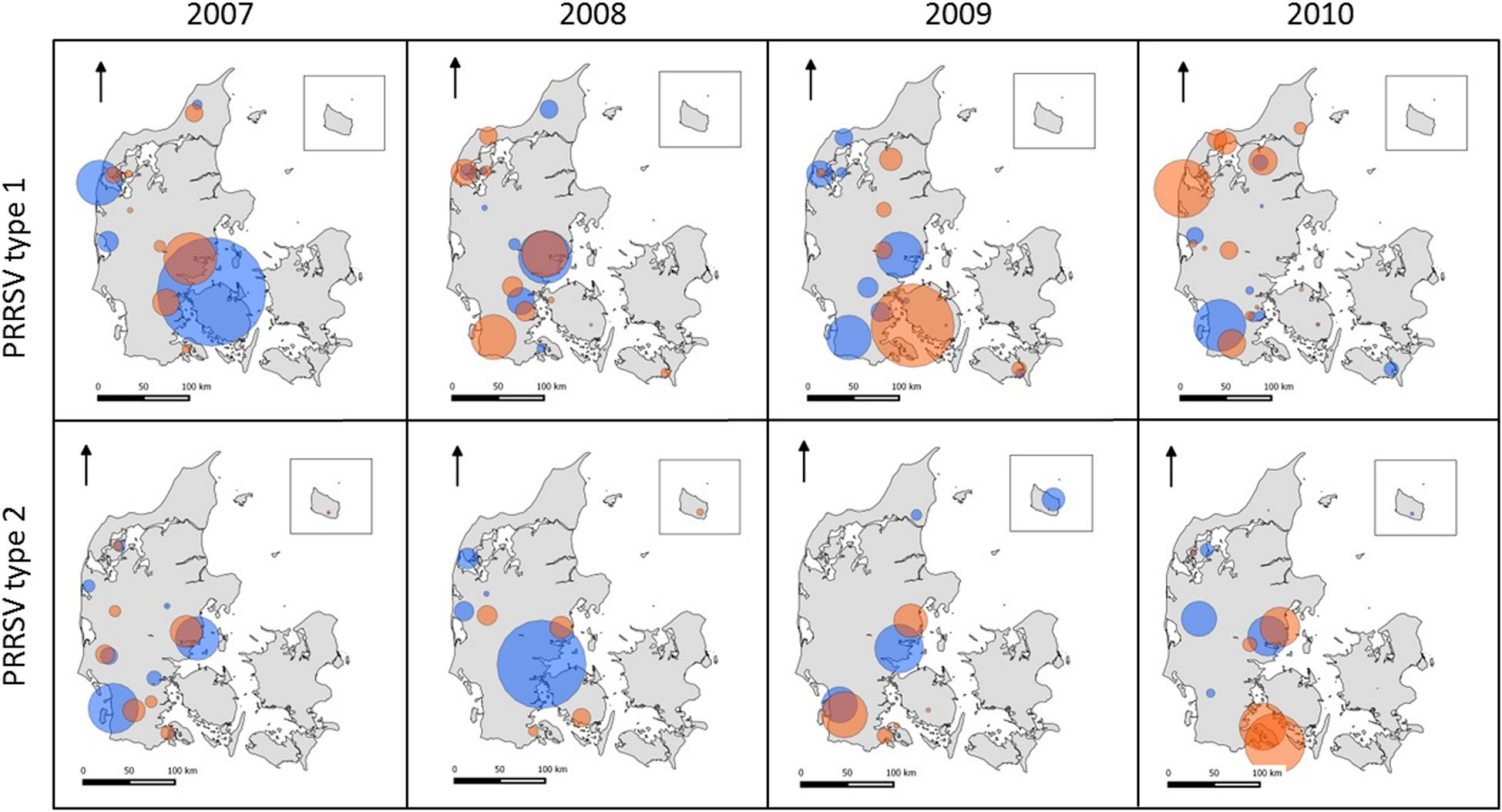
Significant spatial clusters for PRRSV in Denmark. The figure represents significant clusters (*p*-value <0.05) of PRRSV type 1 and type 2 seropositive swine herds in Denmark biannually between 2007 and 2010 for a maximum spatial window size of 25 % of the population at risk. The orange and blue clusters represent the first and second 6-month period in each year, respectively

The significant spatial clusters of PRRSV type 1 and 2 are shown in Fig. 6. The descriptive statistics of these significant clusters are presented in Additional file 1. Increasing the maximum spatial window size from 5 to 15 % of the population at risk resulted in the aggregation of two or more secondary clusters for some 6-month periods. For higher percentages of populations at risk, the number and size of clusters did not change (results not shown). Several clusters were found for each 6-month period. The spatiotemporal pattern for PRRSV type 1 clusters changed over time, except for those located in the northwest of Jutland. Similarly, the locations and sizes of PRRSV type 1 clusters also altered over time from January 2007 to December 2010. In this case, there was a constant cluster in the central eastern part of Jutland.

## Discussion

This is the first study to use surveillance data from laboratory submissions to describe the occurrence of PRRSV in Denmark. The use of laboratory submission records was essential in order to gather previous information and assess the spatial distribution of PRRSV seropositive herds. Such information might be used to evaluate the efficiency of control strategies implemented on a local or regional basis. Using laboratory submission data from a surveillance program might help to identify and record new PRRSV cases in a more reliable way than other sources of information.

The frequency of testing and the type of serological test requested depends on the Danish herd status (SPF or non SPF) and the purpose (PRRSV surveillance or diagnostic). For example, if the objective is to detect infection early, IPMA is normally requested, because high IPMA values are indicative of recent infection as ELISA titers tend to persist for a longer time period [19]. Animals can also be tested for trading purposes, to maintain/gain an SPF certificate, and prior to being introduced in a farm after sanitation procedures. Different reasons for testing might explain the variation in frequency of laboratory submissions in both herd types over the study period. It is our general assumption that herds submitting samples for surveillance or diagnosis have a higher health status compared to those herds that never submit samples (personal communication, C.S. Kristensen, 2014). Therefore, the overall seroprevalence of PRRSV in Danish swine herds may be underestimated based on the submission data used in the present study.

The serological tests used in this study do not differentiate between antibodies from the naturally infected pigs, and those that have been vaccinated against PRRS. However, it is reasonable to assume that an observed seroconversion will be related to a preceding natural infection with PRRSV of homologous type, since vaccination is unlikely in a PRRSV-negative herd. In this study, we therefore argue that an observed seroconversion must initially have been caused by a natural infection, yet we are aware that we might have been measuring vaccine antibodies at the time of sampling.

Herds were classified as seropositive for PRRSV type 1 or type 2 based on the number of seropositive samples per submission. Individual blood samples tested by double ELISAs and IPMAs were classified based on the latest serological results, in order to focus on the most recent PRRSV status [33] demonstrated that high titers in IPMA are indicative of new PRRSV infections. In addition, [19] demonstrated that detection in the IPMA decreases after 3–4 months post infection, therefore making ELISA a more sensitive test to detect late immune responses. In our study, seroprevalence and the seroconversion rate were calculated based on both types of serological tests in order to have the maximum information available over time for each herd.

In this study, the seroprevalence was calculated on a monthly basis to describe the occurrence of PRRSV type 1 and 2 in Denmark. Variation in the seroprevalence for both types might be explained by variation in the number of herds tested per month and the SPF status. Figure 3 indicates an overall decrease in prevalence for both PRRSV strains in both herd types. A recent study by [18] based on information available from the SPF system database estimated that 65 % of sow herds and 60 % of finisher herds in Denmark are PRRSV negative. In our study, these types of herds were classed as production herds, and our results agreed with these findings. The high biosecurity and monthly surveillance of the breeding herds might explain the relatively constant seroconversion rate in Fig. 5.

The information available to us from the SPF system database only provided the herd status on the 31 December of each year. It is therefore unknown whether these herds were under sanitation controls, or if their SPF status changed over time, resulting in possible variation in the frequency of PRRSV testing, which in turn could have influenced the number of new PRRSV seropositive herds. For example, if the SPF status of a PRRSV seropositive breeding herd changed, the herd would be included as a different type in the analysis. This would result in an unknown PRRS status for a period of time, and classification as newly PRRS seropositive when gaining the same SPF status. This happened when a red herd in the SPF database (i.e. a breeding herd) lost their SPF status for a period of time. It was not possible to establish the seroconversion rate for production herds due to the long period of time between consecutive laboratory submissions.

The relative risk distribution maps changed over time as a consequence of the changes in the seroprevalence. The general decline in the extent of areas with higher relative risk for both PRRSV types followed the same trend as observed for the seroprevalence.

## Conclusions

This study described the occurrence of PRRSV in Denmark from 2007 to 2010, based on laboratory submission data. PRRSV type 1 seroprevalence was consistently higher than type 2 seroprevalence in both production and breeding herds. The relative risk maps showed changes in the spatial distribution of both PRRSV types over time. Significant spatial clusters were consistently found in Denmark, suggesting that PRRSV is endemic in these areas. Furthermore, relative risk distribution maps revealed different patterns over time as a consequence of the changes on the seroprevalence.

Our findings might help decision makers to re-evaluate their conclusions on the spread of the disease and assess the efficiency of the implemented control strategies.

## Additional file

Additional file 1:
**Descriptive statistics of significant spatial clusters for PRRSV type 1 and 2.** (DOCX 78 kb)

## Abbreviations

ELISA: enzyme-linked immunosorbent assay
IPMA: immunoperoxidase monolayer assay
PRRS: porcine reproductive and respiratory syndrome
PRRSV: porcine reproductive and respiratory syndrome virus
SPF: specific pathogen free system
