# Supplementary material for: Spatial analysis and temporal trends of porcine reproductive and respiratory syndrome in Denmark from 2007 to 2010 based on laboratory submission data
Source: BMC Vet Res. 2015 Dec 21;11:303. doi: 10.1186/s12917-015-0617-0 (PMC4687366; doi:10.1186/s12917-015-0617-0)
Supplement: Additional file 1: — Descriptive statistics of significant spatial clusters for PRRSV type 1 and 2. (DOCX 78 kb) [file 12917_2015_617_MOESM1_ESM.docx]

**Appendix A: Statistical significant clusters description.**

Statistically significant clusters (p<0.05) of PRRSV type 1 seropositive swine herds in Denmark biannually between 2007 and 2010. For each cluster, the type of cluster and radius are given along with descriptive statistics of the number PRRSV type 1 seropositive herds in the cluster (observed and expected), cluster significance (p-value), Log Likelihood Ratio (LLR) and Relative Risk (RR) for a maximum spatial window size of 25% of the population at risk.

| **No. of PRRSV type 1 seropositive herds in cluster** | | | | | | | |
| --- | --- | --- | --- | --- | --- | --- | --- |
| **Time** | **Cluster type** | **Radius (m)** | **Observed** | **Expected** | **p-value** | **LLR** | **RR** |
| Jan - Jun 2007 | Primary | 60,166 | 185 | 118 | <0.01 | 27.89 | 1.86 |
|  | Secondary | 25,213 | 36 | 11 | <0.01 | 26.52 | 3.41 |
|  | Secondary | 5,198 | 14 | 3 | <0.01 | 19.87 | 4.99 |
|  | Secondary | 812 | 9 | 2 | <0.01 | 14.97 | 5.31 |
|  | Secondary | 11,522 | 14 | 4 | 0.02 | 11.42 | 3.56 |
| Jul - Dec 2007 | Primary | 29,209 | 80 | 31 | <0.01 | 40.36 | 2.86 |
|  | Secondary | 14,765 | 50 | 17 | <0.01 | 30,31 | 3,08 |
|  | Secondary | 9,014 | 18 | 4 | <0.01 | 19.11 | 4.22 |
|  | Secondary | 4,137 | 10 | 2 | <0.01 | 16.67 | 5.34 |
|  | Secondary | 4,615 | 10 | 2 | <0.01 | 16.67 | 5.34 |
|  | Secondary | 2,880 | 9 | 2 | <0.01 | 15.00 | 5.33 |
|  | Secondary | 9,798 | 17 | 5 | <0.01 | 13.52 | 3.52 |
|  | Secondary | 6,251 | 7 | 1 | 0.02 | 11.66 | 5.31 |
| Jan - Jun 2008 | Primary | 11,710 | 19 | 4 | <0.01 | 30.66 | 5.09 |
|  | Secondary | 49,331 | 176 | 109 | <0.01 | 29.64 | 1.90 |
|  | Secondary | 2,880 | 7 | 1 | 0.02 | 11.23 | 5.00 |
|  | Secondary | 10,860 | 12 | 3 | 0.03 | 11.19 | 3.77 |
| Jul - Dec 2008 | Primary | 10,556 | 47 | 16 | <0.01 | 31.70 | 3.18 |
|  | Secondary | 14,589 | 32 | 9 | <0.01 | 27.70 | 3.63 |
|  | Secondary | 25,531 | 61 | 28 | <0.01 | 21.53 | 2.32 |
|  | Secondary | 4,974 | 13 | 3 | <0.01 | 15.03 | 4.22 |
|  | Secondary | 11,158 | 12 | 3 | <0.01 | 13.60 | 4.17 |
|  | Secondary | 1,392 | 8 | 2 | <0.01 | 12.56 | 4.84 |
|  | Secondary | 24,628 | 22 | 8 | 0.02 | 11.34 | 2.70 |
|  | Secondary | 1,604 | 9 | 2 | 0.02 | 11.12 | 4.36 |
|  | Secondary | 3,251 | 7 | 1 | 0.04 | 10.99 | 4,83 |
|  | Secondary | 5,827 | 7 | 1 | 0.04 | 10.99 | 4.83 |
|  | Secondary | 9,852 | 7 | 1 | 0.04 | 10.99 | 4.83 |
| Jan - Jun 2009 | Primary | 8,577 | 44 | 16 | <0.01 | 25.92 | 2.97 |
|  | Secondary | 27,205 | 58 | 27 | <0.01 | 19.43 | 2.28 |
|  | Secondary | 30,351 | 37 | 14 | <0.01 | 19.12 | 2.76 |
|  | Secondary | 22,482 | 29 | 10 | <0.01 | 17.76 | 3.00 |
|  | Secondary | 3,247 | 11 | 2 | <0.01 | 14.57 | 4.66 |
|  | Secondary | 4,664 | 11 | 2 | <0.01 | 14.57 | 4.66 |
|  | Secondary | 9,232 | 14 | 4 | <0.01 | 14.02 | 3.96 |
|  | Secondary | 1,506 | 8 | 2 | <0.01 | 12.93 | 5.06 |
|  | Secondary | 1,392 | 7 | 1 | 0.03 | 11.31 | 5.05 |
|  | Secondary | 4,608 | 7 | 1 | 0.03 | 11.31 | 5.05 |
| Jul - Dec 2009 | Primary | 12,471 | 26 | 6 | <0.01 | 26.92 | 4.30 |
|  | Secondary | 45,822 | 135 | 79 | <0.01 | 25.63 | 1.98 |
|  | Secondary | 2,106 | 12 | 2 | <0.01 | 20.70 | 5.67 |
|  | Secondary | 8,104 | 15 | 3 | <0.01 | 20.14 | 5.03 |
|  | Secondary | 7,933 | 14 | 3 | <0.01 | 15.41 | 4.42 |
|  | Secondary | 9,449 | 7 | 1 | <0.01 | 12.05 | 5.62 |
|  | Secondary | 5,096 | 6 | 1 | 0.03 | 10.32 | 5.61 |
| Jan - Jun 2010 | Primary | 8,798 | 19 | 5 | <0.01 | 19.64 | 4.20 |
|  | Secondary | 9,280 | 18 | 4 | <0.01 | 18.21 | 4.14 |
|  | Secondary | 1,314 | 10 | 2 | <0.01 | 16.88 | 5.45 |
|  | Secondary | 2,106 | 10 | 2 | <0.01 | 16.88 | 5.45 |
|  | Secondary | 5,513 | 13 | 3 | <0.01 | 16.49 | 4.75 |
|  | Secondary | 5,266 | 15 | 4 | <0.01 | 16.41 | 4.33 |
|  | Secondary | 1,392 | 8 | 1 | <0.01 | 13.49 | 5.44 |
|  | Secondary | 1,892 | 8 | 1 | <0.01 | 13.49 | 5.44 |
|  | Secondary | 28,701 | 33 | 14 | <0.01 | 12.74 | 2.42 |
|  | Secondary | 703 | 7 | 1 | 0.01 | 11.80 | 5.43 |
|  | Secondary | 4,308 | 7 | 1 | 0.01 | 11.80 | 5.43 |
|  | Secondary | 7,933 | 12 | 3 | 0.02 | 10.99 | 3.86 |
| Jul - Dec 2010 | Primary | 15,642 | 26 | 7 | <0.01 | 22.78 | 4.10 |
|  | Secondary | 2,106 | 12 | 2 | <0.01 | 22.78 | 6.76 |
|  | Secondary | 2,030 | 10 | 2 | <0.01 | 18.96 | 6.73 |
|  | Secondary | 4,004 | 9 | 1 | <0.01 | 17.05 | 6.72 |
|  | Secondary | 32,251 | 34 | 13 | <0.01 | 15.98 | 2.79 |
|  | Secondary | 15,340 | 17 | 4 | <0.01 | 14.60 | 3.99 |
|  | Secondary | 10,709 | 11 | 2 | <0.01 | 14.07 | 5.29 |
|  | Secondary | 12,625 | 9 | 2 | <0.01 | 13.96 | 6.04 |
|  | Secondary | 9,771 | 14 | 3 | <0.01 | 13.46 | 4.31 |
|  | Secondary | 4,923 | 11 | 2 | <0.01 | 12.81 | 4.94 |
|  | Secondary | 6,747 | 11 | 2 | <0.01 | 12.81 | 4.94 |
|  | Secondary | 1,957 | 6 | 1 | 0.02 | 11.35 | 6.67 |
|  | Secondary | 2,125 | 6 | 1 | 0.02 | 11.35 | 6.67 |
|  | Secondary | 2,435 | 6 | 1 | 0.02 | 11.35 | 6.67 |

Statistically significant clusters (p<0.05) of PRRSV type 2 seropositive swine herds in Denmark biannually between 2007 and 2010. For each cluster, the type of cluster and radius are given along with descriptive statistics of the number PRRSV type 2 seropositive herds in the cluster (observed and expected), cluster significance (p-value), Log Likelihood Ratio (LLR) and Relative Risk (RR) for a maximum spatial window size of 25% of the population at risk.

| **No. of PRRSV type 2 seropositive herds in cluster** | | | | | | | |
| --- | --- | --- | --- | --- | --- | --- | --- |
| **Time** | **Cluster type** | **Radius (m)** | **Observed** | **Expected** | **p-value** | **LLR** | **RR** |
| Jan - Jun 2007 | Primary | 24,144 | 51 | 14 | <0.01 | 40.45 | 3.95 |
|  | Secondary | 6,511 | 29 | 6 | <0.01 | 36.53 | 5.41 |
|  | Secondary | 9,404 | 20 | 4 | <0.01 | 26.66 | 5.57 |
|  | Secondary | 4,170 | 10 | 1 | <0.01 | 19.09 | 6.82 |
|  | Secondary | 8,053 | 12 | 2 | <0.01 | 14.57 | 5.13 |
|  | Secondary | 27,483 | 22 | 7 | <0.01 | 13.68 | 3.25 |
|  | Secondary | 803 | 7 | 1 | <0.01 | 13.34 | 6.78 |
|  | Secondary | 0 | 6 | 1 | 0.02 | 11.43 | 6.76 |
|  | Secondary | 3,124 | 6 | 1 | 0.02 | 11.43 | 6.76 |
|  | Secondary | 6,774 | 6 | 1 | 0.02 | 11.43 | 6.76 |
| Jul - Dec 2007 | Primary | 17,702 | 28 | 7 | <0.01 | 22.04 | 4.05 |
|  | Secondary | 5,270 | 14 | 2 | <0.01 | 18.33 | 5.99 |
|  | Secondary | 10,407 | 14 | 3 | <0.01 | 17.31 | 5.70 |
|  | Secondary | 7,590 | 11 | 2 | <0.01 | 16.47 | 6.67 |
|  | Secondary | 449 | 7 | 1 | <0.01 | 14.83 | 8.40 |
|  | Secondary | 1,004 | 7 | 1 | <0.01 | 14.83 | 8.40 |
|  | Secondary | 1,767 | 7 | 1 | <0.01 | 14.83 | 8.40 |
|  | Secondary | 12,322 | 9 | 2 | 0.01 | 11.57 | 5.84 |
|  | Secondary | 6,509 | 9 | 2 | 0.01 | 11.57 | 5.84 |
| Jan - Jun 2008 | Primary | 2,185 | 17 | 2 | <0.01 | 32.47 | 8.95 |
|  | Secondary | 10,502 | 14 | 2 | <0.01 | 17.33 | 6.01 |
|  | Secondary | 10,965 | 13 | 2 | <0.01 | 17.27 | 6.40 |
|  | Secondary | 3,680 | 8 | 1 | <0.01 | 15.04 | 8.62 |
|  | Secondary | 7,063 | 11 | 2 | 0.01 | 12.11 | 5.37 |
|  | Secondary | 2,111 | 5 | 1 | 0.02 | 11.27 | 9.60 |
|  | Secondary | 26,219 | 25 | 10 | 0.03 | 10.53 | 2.76 |
|  | Secondary | 11,752 | 11 | 2 | 0.04 | 10.27 | 4.66 |
|  | Secondary | 12,411 | 9 | 2 | 0.04 | 10.14 | 5.46 |
|  | Secondary | 6,073 | 7 | 1 | 0.04 | 10.02 | 6.76 |
| Jul - Dec 2008 | Primary | 12,430 | 31 | 6 | <0.01 | 36.35 | 5.97 |
|  | Secondary | 3,526 | 10 | 1 | <0.01 | 22.73 | 9.88 |
|  | Secondary | 5,362 | 10 | 1 | <0.01 | 14.78 | 7.05 |
|  | Secondary | 10,116 | 10 | 2 | <0.01 | 13.72 | 6.58 |
|  | Secondary | 10,976 | 10 | 2 | 0.02 | 10.55 | 5.18 |
| Jan - Jun 2009 | Primary | 2,432 | 12 | 1 | <0.01 | 29.63 | 12.11 |
|  | Secondary | 5,694 | 11 | 1 | <0.01 | 27.13 | 12.06 |
|  | Secondary | 19,351 | 16 | 2 | <0.01 | 21.53 | 7.01 |
|  | Secondary | 11,876 | 11 | 1 | <0.01 | 18.78 | 8.83 |
|  | Secondary | 1,224 | 5 | 0 | 0.01 | 12.27 | 11.76 |
|  | Secondary | 26,421 | 24 | 9 | 0.02 | 10.68 | 2.91 |
| Jul - Dec 2009 | Primary | 18,235 | 32 | 6 | <0.01 | 34.95 | 5.85 |
|  | Secondary | 668 | 8 | 1 | <0.01 | 19.18 | 11.18 |
|  | Secondary | 8,221 | 12 | 2 | <0.01 | 16.15 | 6.80 |
|  | Secondary | 2,342 | 8 | 1 | <0.01 | 16.14 | 9.93 |
|  | Secondary | 3,145 | 5 | 0 | 0.01 | 11.96 | 11.04 |
|  | Secondary | 24,891 | 18 | 6 | 0.02 | 10.42 | 3.38 |
| Jan - Jun 2010 | Primary | 3,437 | 13 | 1 | <0.01 | 31.22 | 11.32 |
|  | Secondary | 22,047 | 37 | 10 | <0.01 | 27.58 | 4.22 |
|  | Secondary | 1,858 | 6 | 1 | <0.01 | 14.32 | 11.01 |
|  | Secondary | 4,582 | 6 | 1 | <0.01 | 14.32 | 11.01 |
|  | Secondary | 7,229 | 8 | 1 | <0.01 | 12.97 | 8.06 |
|  | Secondary | 19,410 | 20 | 7 | 0.05 | 9.80 | 3.04 |
| Jul – Dec  2010 | Primary | 21,883 | 37 | 8 | <0.01 | 37.40 | 5.40 |
|  | Secondary | 3,552 | 12 | 1 | <0.01 | 28.01 | 10.56 |
|  | Secondary | 790 | 10 | 1 | <0.01 | 23.30 | 10.48 |
|  | Secondary | 25,883 | 37 | 13 | <0.01 | 18.80 | 3.18 |
|  | Secondary | 33,150 | 32 | 10 | <0.01 | 18.18 | 3.39 |
|  | Secondary | 575 | 7 | 1 | <0.01 | 16.27 | 10.36 |
|  | Secondary | 3,042 | 6 | 1 | <0.01 | 13.94 | 10.32 |
|  | Secondary | 7,817 | 10 | 2 | 0.01 | 11.74 | 5.80 |
